# *Trichoderma hamatum* Increases Productivity, Glucosinolate Content and Antioxidant Potential of Different Leafy *Brassica* Vegetables

**DOI:** 10.3390/plants10112449

**Published:** 2021-11-12

**Authors:** Pablo Velasco, Víctor Manuel Rodríguez, Pilar Soengas, Jorge Poveda

**Affiliations:** 1 Misión Biológica de Galicia (MBG-CSIC), 36143 Pontevedra, Spain; pvelasco@mbg.csic.es (P.V.); vmrodriguez@mbg.csic.es (V.M.R.); psoengas@mbg.csic.es (P.S.); 2 Institute for Multidisciplinary Research in Applied Biology (IMAB), Campus Arrosadía, Universidad Pública de Navarra, 31006 Pamplona, Spain

**Keywords:** *Trichoderma*, cabbage, turnip greens, phenols, aliphatic glucosinolates

## Abstract

*Brassica* crops include important vegetables known as “superfoods” due to the content of phytochemicals of great interest to human health, such as glucosinolates (GSLs) and antioxidant compounds. On the other hand, *Trichoderma* is a genus of filamentous fungi that includes several species described as biostimulants and/or biological control agents in agriculture. In a previous work, an endophytic strain of *Trichoderma hamatum* was isolated from kale roots (*Brassica oleracea* var. *acephala*), describing its ability to induce systemic resistance in its host plant. In the present work, some of the main leafy *Brassica* crops (kale, cabbage, leaf rape and turnip greens) have been root-inoculated with *T. hamatum*, having the aim to verify the possible capacity of the fungus as a biostimulant in productivity as well as the foliar content of GSLs and its antioxidant potential, in order to improve these “superfoods”. The results reported, for the first time, an increase in the productivity of kale (55%), cabbage (36%) and turnip greens (46%) by *T. hamatum* root inoculation. Furthermore, fungal inoculation reported a significant increase in the content of total GSLs in cabbage and turnip greens, mainly of the GSLs sinigrin and gluconapin, respectively, along with an increase in their antioxidant capacity. Therefore, *T. hamatum* could be a good agricultural biostimulant in leafy *Brassica* crops, increasing the content of GSLs and antioxidant potential of great food and health interest.

## 1. Introduction

*Brassica* genus, belonging to the Brassicaceae family (also known as Cruciferae), includes several crops with a high economic interest, cultivated for their edible roots, leaves, stems, buds, flowers, mustard and oilseeds [1]. The principal vegetable species belonging to the *Brassica* genus are *Brassica napus* (i.e., rapeseed and leaf rape), *Brassica oleracea* (i.e., broccoli, cabbage, cauliflower, kale, Brussels sprouts, etc.), *Brassica rapa* (i.e., turnip, Chinese cabbage and pak choi), *Brassica juncea* and *Brassica carinata* (mustards) [2].

Crops belonging to the *Brassica* genus are among the 10 most economically important vegetable crops in global agriculture and markets [2]. They are mainly cultivated in temperate regions of the northern hemisphere, such as areas of Europe, the Mediterranean area, Southwestern and Central Asia, China and Japan, and North America [2]. In 2019, the Food and Agriculture Organization of the United Nations (FAO) reported a global production of *Brassica* crops close to 170 million tons, being 27 million tons of ‘cauliflowers and broccoli’ in more than 1.4 million ha, 70 million tons of ‘rapeseed’ in more than 34 million ha, and about 70 million tons of ‘cabbages and other *Brassica* crops’ from almost 2.5 million ha in more than 150 countries [3]. Some of the main leafy *Brassica* crops include kale (*B. oleracea* var. *acephala*), cabbage (*B. oleracea* var. *capitata*), leaf rape (*B. napus* var. *napus*), and turnip tops and greens (*B. rapa* var. *rapa*) [4], which are the crops used in the present work.

Nowadays, consumers are demanding products (leaves, flower primordia, roots, oils, etc.) rich in nutrients for optimal health benefits. In this respect, the popularity of *Brassica* products is increasing because of their nutritional value and anticancer, antioxidant and anti-inflammatory properties [5]. Nutritionally, these vegetables are low-fat, have a high vitamin (C and E) content and contain minerals (P, S, Cl, Ca, Fe, Sr, K, Cr, Mn, Se and Zn) and fiber [5]. In addition, they contain important phytochemicals that are beneficial for human health, such as glucosinolates, anthocyanins, flavonoids, terpenes, S-methyl cysteine sulfoxide, coumarins and other small compounds [5].

Glucosinolates (GSLs) are a group of secondary plant metabolites found mainly in the order Brassicales and in the Brassicaceae family [6]. These phytochemicals are sulfur compounds derived from amino acids [7], whose main biological activity in plants is to act as defense against pathogens and pests [8,9]. Inside the cells, GSLs are chemically stable, but upon cell disruption due to tissue damage, GSLs are exposed to the activity of myrosinase and associated proteins, resulting in the so-called GHPs (glucosinolate hydrolysis products) [7].

*Brassica* vegetables contain about 50 different GSLs, although some species can present 15 different GSLs, with three to four of them being predominant (sinigrin, glucoraphanin, gluconapin and glucobrassicin) [10]. The highest concentration of GSLs is found in siliques and seeds, followed by leaves (approximately 1% of dry weight) [10]. Most of the studies carried out to date on GSLs in food have focused on *Brassica* vegetables, due to their importance in the human diet and their possible effects on disease prevention [10]. Among the effects that the consumption of GSLs has on human health, they stand out for their antimicrobial, antioxidant, anti-inflammatory, cholinesterase inhibitory, cytotoxic/anti-cancer, controlling blood sugar levels, antiglycation, suppression of mutagenicity, antifibrotic, antispasmodic, bone formation and parasiticidal activities [11].

In addition, *Brassica* vegetables have a higher antioxidant potential than other vegetable crops, mainly due to a higher content of phenolic compounds [12]. The antioxidant potential of *Brassica* vegetables adds another characteristic of interest for its use as “super food”, providing bioactive compounds that reduce oxidative stress and prevent the appearance of cancer, cardiovascular or neurological diseases [12,13].

In order to increase the productivity of *Brassica* crops and improve its GSL content and antioxidant capacity, different strategies can be considered, including symbiosis with endophytic fungi. *Trichoderma* is a genus of filamentous fungi that inhabit the rhizosphere and can behave as endophyte by colonizing the roots, without penetrating even the vascular bundles [14]. *Trichoderma* includes several species widely studied and used as beneficial microorganisms in agriculture, primarily as biological control agents, due to different mechanisms of action, such as mycoparasitism, antibiosis, space and nutrient competition, modulation of plant local and systemic resistance, mediated tolerance to abiotic stresses and plant growth promotion [15]. The ability of *Trichoderma* to promote plant growth and productivity in crops is a consequence of the production of different secondary metabolites (harzianic acid, 6-pentyl-a-pyrone) and phytohormones (auxins, cytokinins), fully known mechanisms in different endophytic fungi [16]. The ability to promote plant growth and yield has been previously described for *Trichoderma* in tomato or vineyard crops [17,18].

Root inoculation of *Brassica* crops with *Trichoderma* has reported interesting results in the last years. In rapeseed (*B. napus*), inoculation with *T. harzianum* supposes an increase in productivity of about 50% [19], which is also reported with the inoculation of *T. parareesei* under drought and salinity conditions at about 160% and 700%, respectively [20]. Similarly, a plant growth promotion and/or yield increment has been observed in butter cabbage (*B. oleracea* var. *acephala*) inoculated with *T. asperelloides* and *T. virens* [21]; in flowering Chinese cabbage (*B. campestris* ssp. *chinensis* var. *utilis*) inoculated with *T. harzianum*, *T. asperellum*, *T. hamatum* and *T. atroviride* [22]; and in pak choi (*B. chinensis*) inoculated with *T. atroviride* and *T. citrinoviride* [23]. Furthermore, the inoculation of *Brassica* crops roots with *Trichoderma* can lead to a reduction in the incidence of diseases by acting as a biological control agent, as has been reported in Chinese cabbage (*Brassica rapa* subsp. *pekinensis*) inoculated with *T. harzianum* against *Plasmodiaphora brassicae* (clubroot disease) [24], or in kale (*B. oleracea* var. *acephala*) inoculated with *T. hamatum* against *Xanthomonas campestris* (black rot) [25].

By colonizing the roots, *Trichoderma* is capable of modulating plant physiology, causing changes in the foliar metabolome. The colonization of maize roots by *T. virens* produces very significant metabolomic changes in the root and aerial part of the plant [26], changes that facilitate the response to different stresses, as has been observed in tomato under drought stress inoculated with *T. harzianum* [27]. Changes in the foliar metabolome have been studied with respect to defense-related secondary metabolites in *Arabidopsis thaliana*-*T. gamsii* system [28] and *T. harzianum*, *T. virens* and *T. asperellum*-olive system [29], although it also refers to metabolites of industrial interest, as in industrial hemp-*T. harzianum* system and the production of cannabidiol [30].

As far as we know, there are no studies conducted on the use of *T. hamatum* to promote plant growth and to increase the GSLs content and antioxidant content in *Brassica* crops. Therefore, the main objective of this work is to use *T. hamatum*, isolated from kale roots, as biostimulant, in order to obtain more productive *Brassica* crops and with better nutraceutical characteristics. To achieve this, *Trichoderma*-root colonization, fresh weight of the aerial plant part, GSL content and its antioxidant activity have been quantified. Therefore, the hypothesis of the work is that *T. hamatum* can improve the cultivation of leafy *Brassica* crops by colonizing their roots, promoting growth of the aerial part and the content of GSLs and antioxidant capacity.

## 2. Results

### 2.1. Effect on the Foliar Yield of Brassica Crops

**Figure 1:**
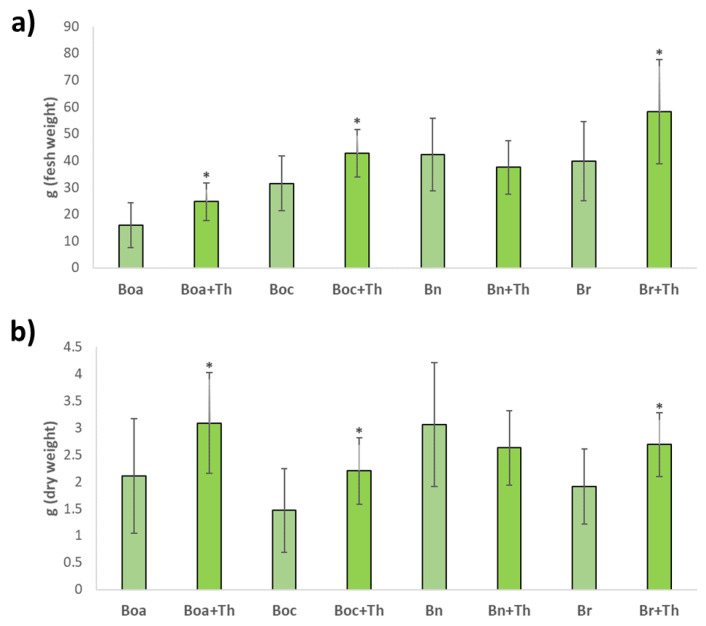
Mean of fresh (**a**) and dry weight (**b**) of kale (Boa), cabbage (Boc), leafy rape (Bn) and leafy turnip (Br) grown in greenhouse conditions. Plants without inoculation and inoculated with *T. hamatum* (+Th) were collected at 8 weeks old and measured their fresh and dry weight of the aerial part. Data are the mean of 10 plants for each crop and condition with the corresponding standard deviation. Student’s *t*-test was performed between uninoculated and *Trichoderma*-inoculated plants. Asterisks denote significant differences at *p* ≤ 0.05 (*).

In leafy *Brassica* crops, the foliar mass refers directly to the yield, as it is the consumed part. Root inoculation with *T. hamatum* represented a significant increase in the fresh and dry biomass of the aerial part of kale, cabbage and turnip greens. Specifically, an increase in the weight of the fresh product of 55% in kale, 36% in cabbage and 46% in turnip greens was reported. However, no significant differences were observed in leaf rape biomass by fungal inoculation (Figure 1).

### 2.2. GSLs Profiles in Brassica Leaves

**Figure 2:**
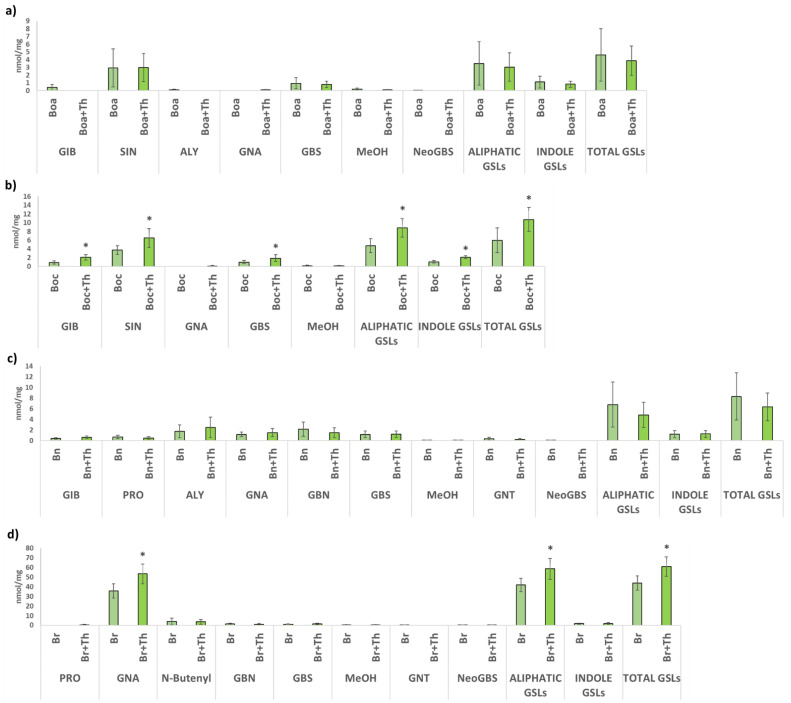
GSLs contents in leaves from kale (Boa) (**a**), cabbage (Boc) (**b**), leafy rape (Bn) (**c**) and leafy turnip (Br) (**d**) without inoculation and inoculated with *T. hamatum* (+Th). GIB: glucoiberin, SIN: sinigrin, PRO: progoitrin, ALY: glucoalyssin, GNA: gluconapin, GBS: glucobrassicin, MeOH: metoxy-glucobrassicin, NeoGBS: neoglucobrassicin, GNT: gluconasturtiin. Data are the mean of 10 plants for each crop and condition with the corresponding standard deviation. Student’s *t*-test was performed between uninoculated and *Trichoderma*-inoculated plants for each GSL. Asterisks denote significant differences at *p* ≤ 0.05 (*).

In both kale and leaf rape, no significant differences were reported in foliar GSL profiles between plants inoculated with *T. hamatum* and without inoculation (Figure 2a,c). In cabbage leaves, root inoculation with *T. hamatum* caused a significant increase in the total content of GSLs (10.74 nmol/mg), both aliphatic GSLs (8.83 nmol/mg) and indole GSLs (2.11 nmol/mg). Within the aliphatic GSLs, a significant increase in glucoiberin (2.01 nmol/mg) and sinigrin (6.49 nmol/mg) was found, while glucobrassicin (1.88 nmol/mg) was the indole GSL stimulated by *T. hamatum* (Figure 2b). In turnip greens, a significant increase in the total content of GSLs (60.84 nmol/mg) and aliphatic GSLs (58.54 nmol/mg) was reported by root inoculation with *T. hamatum*. Specifically, this is due to the significant increase in the content of the aliphatic GSL gluconapin (53.23 nmol/mg) (Figure 2d).

### 2.3. Antioxidant Potential in Brassica Leaves

**Figure 3:**
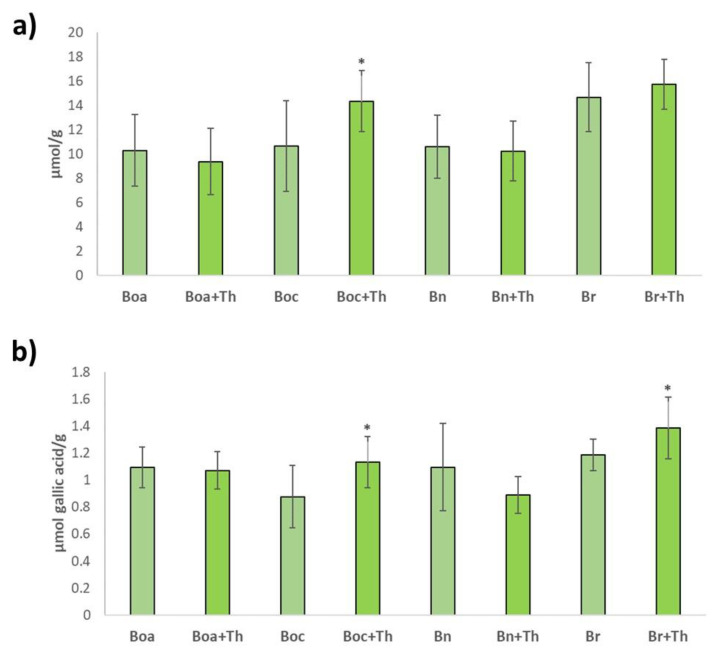
Antioxidant activity (ABTS^+^) (**a**) and phenolic content (Folin reagent) (**b**) in leaves from kale (Boa), cabbage (Boc), leafy rape (Bn) and leafy turnip (Br) without inoculation and inoculated with *T. hamatum* (+Th). Data are the mean of 10 plants for each crop and condition with the corresponding standard deviation. Student’s *t*-test was performed. Asterisks denote significant differences at *p* ≤ 0.05 (*).

In kale, leaf rape and turnip greens leaves, no significant differences were quantified in antioxidant activity (ABTS oxidation) between uninoculated and root-inoculated plants with *T. hamatum*. However, significant differences were reported in the case of cabbage plants (14.36 µmol/g) (Figure 3a). Regarding the phenolic content (measured by Folin reagent), no significant differences were quantified between the kale and leaf rape plants without inoculation and inoculated with the fungus. On the contrary, in cabbage (1.13 µmol gallic acid/g) and turnip greens (1.38 µmol gallic acid/g) plant roots inoculated with *T. hamatum*, significantly higher levels of phenolic compounds were observed in their leaves (Figure 3b).

### 2.4. Trichoderma-Roots Colonization

**Figure 4:**
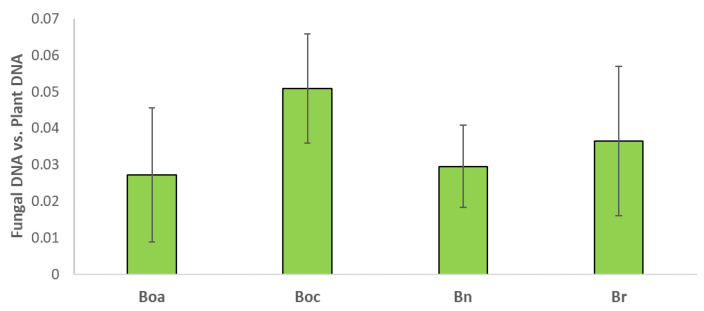
Measurements of *Brassica*-root colonization by *Trichoderma* by qPCR in kale (Boa), cabbage (Boc), leafy rape (Bn) and leafy turnip (Br). Values are the proportion of fungal DNA vs. plant DNA means of three root pools (three plants each one) with the corresponding standard deviations. One-way analysis of variance (ANOVA) was performed, followed by the Tukey’s test. No significant differences (*p* < 0.05) were found.

Regarding the root colonization of *Brassica* plants by *Trichoderma*, no significant differences were quantified in the levels of root colonization between the different leafy crops (Figure 4). However, on average, the roots of *B. oleracea* var. *capitata* and *B. rapa* show a higher rate of colonization by *T. hamatum* (0.051 and 0.037, respectively) than the roots of *B. oleracea* var. *acephala* and *B. napus* (0.027 and 0.029, respectively).

## 3. Discussion

In a previous study, it was reported that *T. hamatum* did not produce a significant increase in the biomass produced by kale plants [25]. However, it involved the activation of a systemic resistance that caused plants to be significantly less affected by the phytopathogenic bacterium *X. campestris* [25]. In our study, we have reported how root inoculation with *T. hamatum* supposes a significant increase in the foliar biomass produced by kale, cabbage and turnip greens. Possibly, the differences observed with respect to the previous study carried out with kale are due to the method of *Trichoderma*-inoculation. In the first study, *Trichoderma* was applied to the roots as mycelium grown on beet pulp, while in this work, the inoculation was carried out as spores. In relation to the results obtained, increases in the biomass of the aerial part in plants root-inoculated with *T. hamatum* have been reported in lettuce [31,32,33], *Vigna mungo* [34] and cacao seedlings [35]. As far as *Brassica* crops are concerned, only kale plants had previously been inoculated with *T. hamatum* [25]. However, in a recent study where Chinese flower cabbage plants were root-inoculated with a formulation of different *Trichoderma* species, including *T. hamatum* together with *T. harzianum*, *T. asperellum* and *T. atroviride*, a significant increase in plant growth and the yield was reported compared to uninoculated plants [22].

The increase in plant growth and GSL content in *Brassica* crops simultaneously by *Trichoderma* inoculations has not been reported previously. Despite this, it has been observed how the irrigation application of *T. harzianum* spores in *B. rapa* subsp. *sylvestris* cv. *esculenta* supposed a higher production of foliar biomass, although it did not report significant changes in the amount of total GSLs accumulated [36]. Regarding the analysis of individual GSLs, it has been determined as the application of *T. atroviride* in rapeseed [37] and of *T. harzianum* and *T. atroviride* in *B. rapa* subsp. *sylvestris* cv. *esculenta* [38] and did not suppose a significant increase in the foliar accumulation of any of them either.

GSLs are widely studied and used compounds in agriculture for their antifungal capacity against a large number of phytopathogenic fungi [9]. Regarding the filamentous fungus *Trichoderma*, it has been described that it requires the action of specific proteins that act on the hydrolysis of GSLs in order to colonize the roots of crucifers [39]. Furthermore, it has been shown that indole GSLs are involved in reducing root colonization by *Trichoderma* in *A. thaliana*. Reporting as in the absence of indole GSLs, *Trichoderma* mostly colonizes the roots, which implies a greater systemic resistance and tolerance against abiotic stresses in *A. thaliana* [40].

In our study, the application of *T. hamatum* in cabbage and turnip greens supposed a significant increase in the content of total GSLs and antioxidant potential in its leaves. This is due to an increase in the content of aliphatic (glucoiberin and sinigrin) and indole GSLs (glucobrassicin) in cabbage and in aliphatic GSLs (gluconapin) in turnip greens. The increase in the content of secondary defense metabolites, such as GSLs and phenols, in both *Brassica* crops could be due to the activation of a systemic resistance by the root colonization of *T. hamatum*, previously described in cucumber [41], tomato [42], geranium [43] or *A. thaliana* [44].

The increase in these GSLs in cabbage and turnip greens represents an important result in the food sector, for the use of *Brassica* leafy crops as nutraceutical foods. Glucoiberin is one of the GSLs of greatest interest in the use of *Brassica* vegetables as anticancer foods [45]. This is due to the fact that the isothiocyanate derived from its hydrolysis (called iberin) is involved in the activity of Phase II enzymes, which are able to conjugate with activated carcinogens and turn them into inactive water-soluble compounds [45]. Sinigrin is present in all *B. oleracea* crops, such as broccoli, brussels sprouts and cabbage. Moreover, it is one of the GSLs with the most benefits for human health, since it has anti-cancer, antibacterial, antifungal, antioxidant, anti-inflammatory, and wound-healing properties [46]. Glucobrassicin is one of the most widely present GSLs in different *Brassica* vegetables, being the natural precursor of recognized anti-cancer and chemo preventive agents, such as indole-3-carbinol and 3,3′-diindolylmethane. These compounds have been widely investigated and have been shown to suppress the proliferation of various cancer cell lines, such as those of breast, colon, prostate and endometrium [47]. Finally, gluconapin is a GSL present in the majority form in leaves of *B. rapa*. Until now, few advantages of its inclusion in the diet have been described, but it is known that it is capable of reducing plasma triglyceride levels, acting as a preventive of postprandial hypertriglyceridemia [48].

Similarly, increasing the antioxidant potential of *Brassica* leaves is an important finding in their use as nutraceutical foods. The ingestion of antioxidants prevents intracellular oxidation, which has been associated with health promotion and the prevention of most degenerative diseases, such as atherosclerosis, cancer, diabetes, arthritis, an improved cardiovascular and neurological health, reduced cancer incidence, increased longevity and lowered overall mortality [49]. In the specific case of phenols, whose content increased in cabbage and turnip greens leaves root-inoculated with *T. hamatum*, they represent an important group of antioxidants to consider in the diet. It is important to note their beneficial effects in the prevention of cancer and cardiovascular, inflammatory and neurodegenerative diseases as well as to their contribution in lowering the risk of depression and cognitive decline during aging [50].

It has been previously described how the beneficial effects of *Trichoderma* on plants are modified according to their levels of fungal-root colonization [39,40]. However, our work reported important differences between the different *Brassica* crops used, without there being a significant difference between the levels of root colonization by *T. hamatum*.

## 4. Materials and Methods

### 4.1. Biological Material

As *Brassica* leafy crops, local populations from the MBG germplasm bank were used: MBG-00063 (*B. napus*), MBG-00163 (*B. rapa*), MBG-00462 (*B. oleracea* var. *acephala*) and MBG-00535 (*B. oleracea* var. *capitata*).

*T. hamatum* was isolated from roots of kale in a previous work with different local populations from Galicia (Northwestern Spain) [25], identified by the sequences ITS (MT641233) and *Tub* (OL389793). The fungus was routinely grown on potato dextrose agar (PDA, Sigma-Aldrich, Madrid, Spain) in the dark at 28 °C. Spores were harvested from 7-day-old PDA dishes, as previously described by [51].

### 4.2. Plant Growth and Trichoderma Inoculation

*Brassica* plants were individually grown from seeds in 5 L pots. The plants were kept under controlled greenhouse conditions until their aerial part was harvested in 8-week-old plants to record fresh and dry weight. The plants were watered one to two times per week, according to the observed needs, always with the same amount of water in all the plants. The plants were grown on a substrate consisting of peat moss (Profi-Substract, Gramoflor, Valencia, Spain) previously treated at 80 °C for 24 h. Exogenous fertilization was not used. Greenhouse conditions were 14 h photoperiod, controlled environmental temperature (12–30 °C) and relative humidity above 80%. A total of 10 plants (in 10 pots) were used for each condition and crop.

Root inoculation with *Trichoderma* was carried out as described by [39] for rapeseed (*B. napus*). In 4-week-old plants, the main root was inoculated with 1 mL of a conidial suspension containing 2 × 10^7^ spore ml^−1^, determined using a hemocytometer.

### 4.3. GSLs Analysis

For GSLS and antioxidant potential analyses, the 4th leaf from the apex of 10 plants were collected in liquid nitrogen and stored at −80 °C until freeze-dried in a lyophilizer (GAMMA 2-16 LSC plus, Christ, Germany). Samples were mechanically milled to a fine powder in a grinder (Janke and Kunkel A10 mill, IKA-Labortechnik, Staufen, Germany) before metabolite extraction. The samples were always kept as 10 independent biological replicates (one per plant).

The analysis of the GSLs profile in the samples was carried out following the methodology described by [52], with some modifications. Twelve mg of freeze-dried *Brassica* leaves powder were mixed with 400 μL 70% (*v*/*v*) methanol preheated to 70 °C, 10 μL of PbAc (0.3 M) and 120 μL ultra-pure water preheated to 70 °C. Before, 20 μL of glucotropaeolin was added as an internal standard. The tubes were shaken in a Microplate incubator (Model OVAN Orbital Midi) at 250 rpm for one hour and centrifuged at 3700 rpm for 12 min. Subsequently, 400 µL of the glucosinolate extracts was pipetted on an ion-exchange column with Sephadex DEAE-A25. By adding of purified sulfatase (E.C. 3.1.6.1, type H-1 from Helix pomatia) (Sigma) solution, desulfation was carried out. Finally, the desulphated GSLs were diluted in 200 µL of ultra-pure water and 200 µL of 70% methanol, keeping frozen for further analyzes.

Using this method, only desulfoglucosinolates were extracted, so a matrix effect is not expected. This fact was further confirmed by spiking with commercial standards. Besides, the percentage of glucosinolate recovery using this method is around 99%.

The chromatographic analyses were carried out on an Ultra-High-Performance Liquid Chromatograph (UHPLC Nexera LC-30AD; Shimadzu, Kyoto, Japan) equipped with a Nexera SIL-30AC injector and one SPDM20A UV/VIS photodiode array detector. The UHPLC column was a X Select ^®^HSS T3 (2.5 µm particle size, 2.1 × 100 mm i.d.) from Waters (Waters Corporation, Milford, MA, USA) protected with a Van Guard pre-column. The oven temperature was set at 35 °C. GSLs were quantified at 229 nm and were separated by using the following method in aqueous acetonitrile, with a flow of 0.5 mL min^−1^: 1.5 min at 100% H_2_O, an 11 min gradient from 5% to 25% (*v*/*v*) acetonitrile, 1.5 min at 25% (*v*/*v*) acetonitrile, a minute gradient from 25% to 0% (*v*/*v*) acetonitrile and a final 3 min at 100% H_2_O. Specific GSLs were identified by comparing retention times and UV spectra with standards. GSLs standards were purchase from Phytoplan (Diehm & Neuberger GmbH, Heidelberg, Germany). Calibration equations were made with at least five data points. Specific GSLs were identified by comparing retention times and UV spectra with standards. GSLs standards were purchased from Phytoplan (Diehm & Neuberger GmbH, Heidelberg, Germany). Calibration equations were made with at least five data points for the glucosinolates glucoiberin (y = 99397x; R^2^ = 0.950), sinigrin (y = 484871x; R^2^ = 0.994), gluconapin (y = 352910x; R^2^ = 0.999), glucobrassicanapin (y = 357893x; R^2^ = 0.997), glucoerucin (y = 276.122x; R^2^ = 0.999), glucobrassicin (y = 869483x; R^2^ = 0.988), gluconasturtiin (y = 342954x; R^2^ = 0.997), progoitrin (y = 398645x; R^2^ = 0.980).

### 4.4. Antioxidant Potential Assays

The antioxidant potential of the foliar tissues was analyzed following the methodology described by [53]. In order to obtain leaf extracts, 1 mL of 80% aqueous methanol was added to 10 mg of the leaf sample and left it in dark for 24 h. Subsequently, the extracts were centrifuged afterward (3700 rpm, 5 min). The antioxidant activity and the content of phenolic compounds were measured in the methanolic extracts using ABTS^+^ assay and Folin reagent, respectively. ABTS^+^ was generated by the oxidation of 7 mM ABTS with 2.45 mM K_2_S_2_O_8_ at room temperature for 16 h. The stock solution of ABTS^+^ was diluted in water to reach an absorbance between 0.8 and 1.0 at 734 nm. Then, 20 μL of each methanolic extract was mixed to 250 mL of the ABTS^+^ solution, and the absorbance was recorded after 30 min of incubation under dark conditions. The results were standardized to Trolox equivalents per gram of dry weight. To quantify total phenolic content, 50 μL of each extract was mixed with 50 μL of 0.5 M Folin reagent. The reaction was neutralized after 5 min with 200 μL of 20% Na_2_CO_3_. After 2 h of incubation in dark conditions, the absorbance of the reaction was measured at 760 nm. 

### 4.5. Quantification of Trichoderma-Root Colonization

The quantification of fungal DNA in *Brassica* roots was performed by qPCR as previously described by [40], with some modifications. DNA was extracted from roots of *Trichoderma* inoculated plants, using the Phire Plant Direct PCR Kit (Termo Fisher Scientifc, Waltham, MA, USA). A mix was prepared in a 10 μL volume using 5 μL of Brilliant SYBR Green QPCR Master Mix (Roche, Penzberg, Germany), 10 ng of DNA, the forward and reverse primers at a final concentration of 100 nM and nuclease-free PCR-grade water to adjust the final volume. Different endogenous genes of each *Brassica* crop and *actin* in *Trichoderma* were used, of which the corresponding first pairs are indicated in Table 1. Amplifications were performed in an ABI PRISM 7000 Sequence Detection System (Applied Biosystems, Foster City, CA, USA) programmed for 40 cycles under the following conditions: denaturation, 95 °C for 15 s; annealing, 60 °C for 1 min; extension, 72 °C for 1 min. Each PCR was performed in triplicate by using the DNA extracted from 3 root pools of 3 plants each, one for each treatment. Cycle threshold values served to calculate the amount of fungal DNA using standard curves. Values of fungus DNA were referred to the amount of kale DNA in every corresponding sample.

From each crop, the whole roots of 9 plants were collected, grouping them into pools of 3 plant-roots. Roots were collected in 8-week-old plants, 4 weeks post-*Trichoderma*-roots inoculation. All root material was washed with water until there was no remaining substrate, immediately frozen with liquid nitrogen, and pulverized with a mortar.

### 4.6. Statistical Analysis

The statistical analysis of the data was carried out with the Statistix 8.0 software. To perform the data normality confirmation analysis, the Shapiro−Wilk test was performed. Student’s *t*-test was used for comparison of means of groups of 10 plants at *p* < 0.05; significant differences are denoted using an asterisk. The group means were represented in columns in the graphs, representing the variance in the form of error bars. In quantification of *Trichoderma*-root colonization, one-way ANOVA using Tukey’s multiple range test at *p* < 0.05 was used for pairwise comparisons; different letters indicate significant differences (*p* < 0.05).

## 5. Conclusions

As conclusions, the root inoculation of *T. hamatum* supposes an increase in the productivity of several leafy *Brassica* crops, being the first report for the fungus in these crops. Furthermore, the fungal application supposes an increase in productivity, GSLs and antioxidant potential (simultaneously) in cabbage (*B. oleracea* var. *capitata*) and turnip greens (*B. rapa* var. *rapa*), representing the first report that relates the *Trichoderma*-inoculation with the increase of GSLs and antioxidant potential in *Brassica* crops. Therefore, *T. hamatum* could be a good agricultural biostimulant in leafy *Brassica* crops, increasing the content of GSLs and antioxidant potential of great food and health interest.

## Figures and Tables

**Table 1:** Primers used in the quantification of *Trichoderma*-root colonization.

Code	Sequence (5′-3′)	Use	References
Act-T-F	ATGGTATGGGTCAGAAGGA	Endogenous *Trichoderma* gene	[40]
Act-T-R	ATGTCAACACGAGCAATGG		
GADPH-Bo-F	TCAGTTGTTGACCTCACGGTT	Endogenous *B. oleracea* gene	[54]
GADPH-Bo-R	CTGTCACCAACGAAGTCAGT		
Tub-Bn-F	TTCAAAGAACATGATGTGTGCC	Endogenous *B. napus* gene	[54]
Tub-Bn-R	CGTTTATCATCTGTTCGTCCAC		
Act-Br-F	TGTGCCAATCTACGAGGGTTT	Endogenous *B. rapa* gene	[55]
Act-Br-R	TTTCCCGCTCGGCTGTTGT		

## Data Availability

Not applicable.
